# Supplementary material for: Maternal thyroid hormone is required to develop the hindbrain vasculature in zebrafish
Source: Commun Biol. 2025 Jul 1;8:960. doi: 10.1038/s42003-025-08404-1 (PMC12216513; doi:10.1038/s42003-025-08404-1)
Supplement: Supplementary file 2 — Supplementary Information [file 42003_2025_8404_MOESM2_ESM.pdf]

Supplementary information

Supplementary Table 1 - Primers for target genes used for qPCR analyzes

Gene	Forward primer (5'-3')	Reverse primer (5'-3')	Product Size (bp)	GenBank accession no.
cxcl12b	ACACACACACACTCGCTCTTG	GGGAGTGTGGAGGAACTTGAT	188	NM_001320414.1
cxcr4a	GGTCAGTCACGACTTCCAGAG	GAGAGGTGCAGACGGTACTTG	150	NM_131882.3
notch1b	CCTGTCATACTGGAGCCACAT	GACTGGGTTGGTGTCACAGTT	149	NM_131302.2
notch3	GCAGTAACAAGAACCGCAGAG	TTAGCCTCTGGGCAGTCTGTA	133	NM_131549.2
nrp1a	ATTACAGCTCCAGGACCCAAC	GCCGTCTCTCACTTCCACATA	105	NM_001040326.1
pax6a	GACAGCCCAATCAAGATGGT	CTGAAGCCTCATTTGGGTCTC	107	NM_131304.1
robo4	GGGGGCACTAGACTGAGAGAC	GAGCGATACAGCACCTCAAAG	170	XM_684163.8
rxrba	ACCGTGTCCATGTCAGGTCT	ACTCCATAGTGCTTGCCAGAG	161	NM_001423448.1
s1pr1	CTCTTCATCCTGCTCCTCCTT	GCTGGTCAGGGTGTAGATCAG	132	NM_131691.3
slit2	GCATCAGTAACCCCTGTCAGA	ATACCGTCCACGCATGTAGAG	169	NM_131735.1
slit3	AGAGGAGCATTTGAGGGTCTC	GTCCAGTTGCAGGTTCTTGAC	192	NM_131736.3
vegfaa	TCTCCTCCATCTGTCTGCTGT	GGATGTACGTGTGCTCGATCT	173	NM_131408.3
vegfab	CATACCGTCCTGTGTGGTTCT	CACCCTGATGACGAAGAGGT	81	NM_001328597.1

Supplementary Table 2 - Primers used for cloning of target sequences and use for *in situ* hybridization riboprobe synthesis, and references of previously published riboprobes.

Gene	Forward primer (5'-3')	Reverse primer (5'-3')	Product Size (bp)	GenBank accession no.
pax6a	AGGCTGTTGGAACTATGCCTC	CGTCGCGTTCTCACTGTAGTC	1380	NM_131304.1
pax8	(Campinho et al., 2014)			
mct8	(Campinho et al., 2014)			

Supplementary Figures

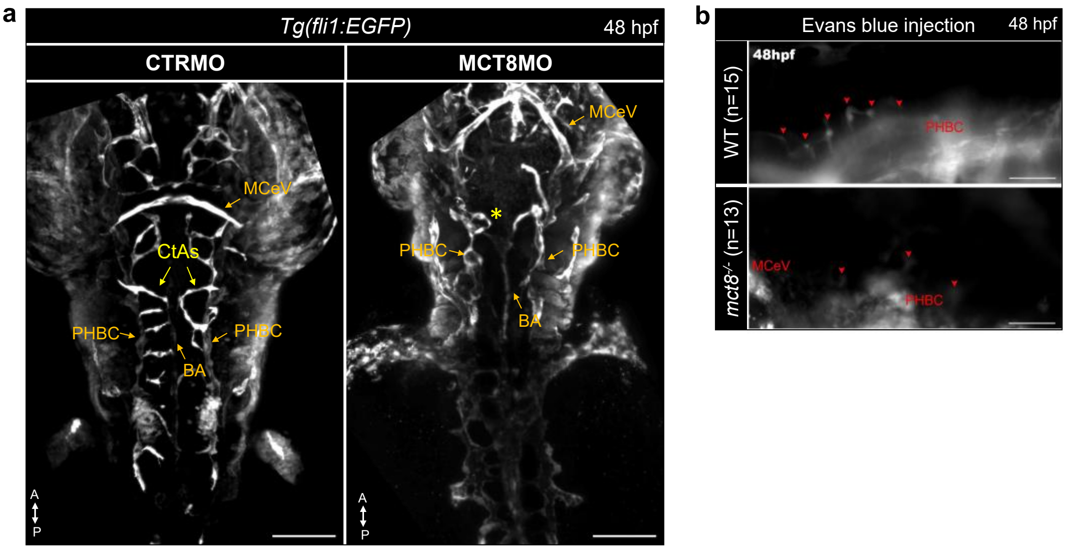

Supplementary Figure 1. Central arteries development is disrupted in *mct8* deficient zebrafish embryos. a) Dorsal view of maximum projection images of *Tg(fli1:EGPF)* zebrafish embryos at 48 hpf immunostained with GFP (endothelial marker, white), showing differences in the number of central arteries (CtAs) present in the hindbrain of MCT8MO embryos compared to CTRMO zebrafish embryos. Asterisks denote reduced CtA sprouting. **b)** Lateral view of wild-type (WT) and *mct8* knockout embryos (*mct8*$^{-/-}$) at 48 hpf previously injected with Evans blue dye. *mct8*$^{-/-}$ zebrafish embryos also present fewer CtAs, similarly to MCT8MO embryos. Red arrowheads indicate CtAs. A – Anterior; BA – Basilar Artery; CtAs – Central Arteries; MCeV – Mid-cerebral Vein; P – Posterior; PHBC – Primordial Hindbrain Channels. Scale bar: 100μm**.**

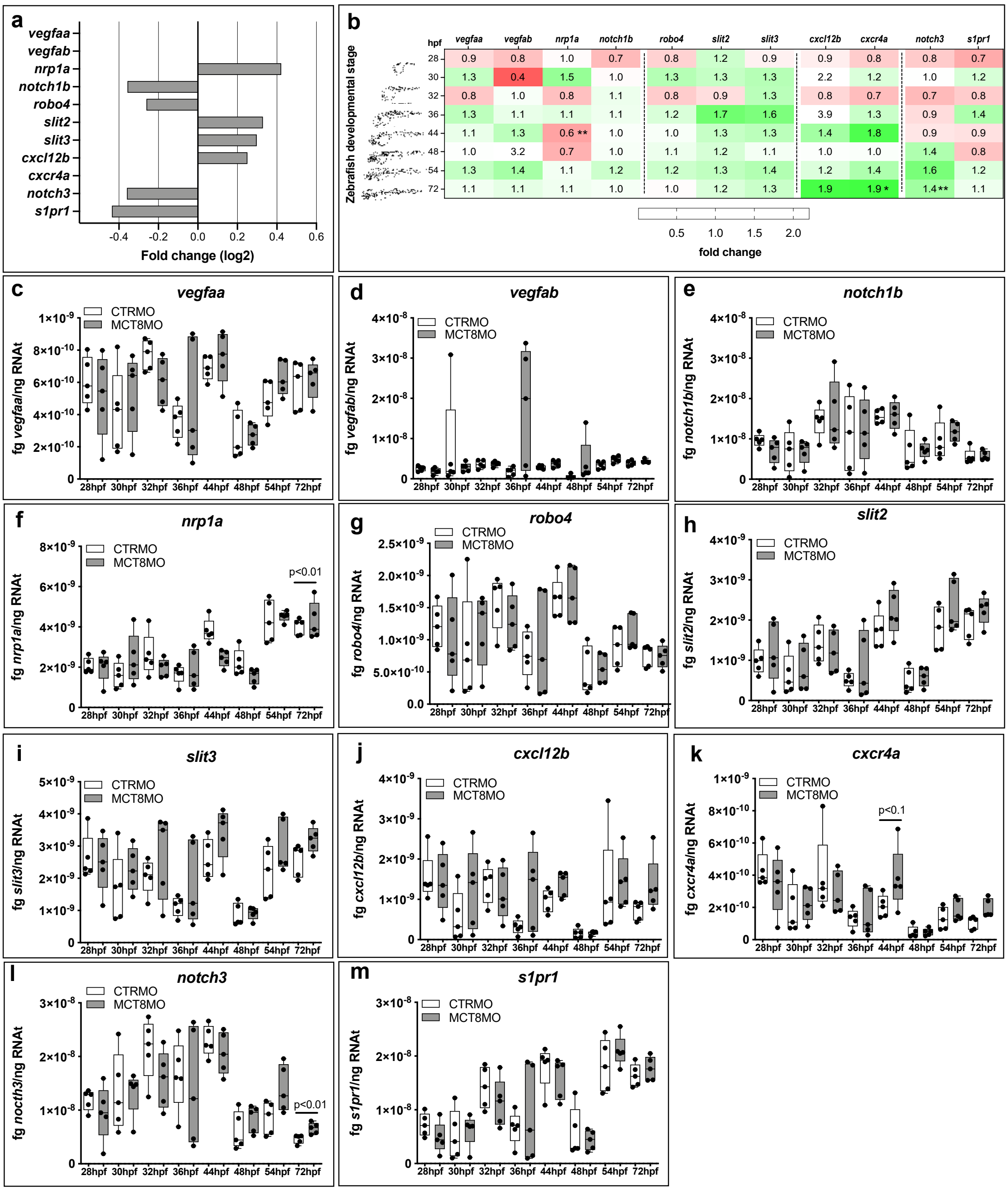

Supplementary Figure 2. Whole-embryo gene expression analysis suggests that MT3 is not involved in regulating angiogenic-related genes. **a)** Genes of interest, involved in angiogenesis, differentially expressed between CTRMO and MCT8MO zebrafish embryos, identified by RNA-Seq expressed as Log2 or fold change (n = 7, p<0.01, FDR<0.0001) (NCBI–BioProjects: PRJNA381309). **b)** Expression of MT3-responsive genes suggests that MT3 are not regulating genes related to the angiogenic process of blood-hindbrain barrier development. Zebrafish developmental stages analyzed by qPCR are depicted by camera lucida drawings adapted from Kimmel et al. Heatmap representation of gene expression levels of *vegfaa*, *vegfab*, *nrp1*, *notch1b*, *robo4*, *slit2*, *slit3*,

rxrcl12b, *rxrc4a*, *notch3*, and *s1pr1*, determined after qPCR in MCT8MO and CTRMO during embryonic development. Data are represented as fold change of MCT8MO expression relative to the CTRMO. Statistical differences were evaluated between MCT8MO and CTRMO for each time point using a t-test after normal distribution was confirmed (D'Agostino & Pearson test). n = 5, except for 36 hpf MCT8MO *vegfab* samples n = 2 (*p<0.05; **p<0.01). **c-m)** Box-and-whiskers plot of gene expression levels determined after qPCR for *vegfaa* **(c)**, *vegfab* **(d)**, *nrp1a* **(e)**, *notch1b* **(f)**, *robo4* **(g)**, *slit2* **(h)**, *slit3* **(i)**, *cxcl12b* **(j)**, *cxcr4a* **(k)**, *notch3* **(l)***,* and *s1pr1* **(m)**. Data is represented as femtograms (fg) of the gene of interest by nanograms (ng) of total RNA used for cDNA preparation. Statistical significance was determined at each time point, comparing gene expression in MCT8MO relative to CTRMO using a t-test after normal distribution was confirmed (D'Agostino & Pearson test). n = 5, except for 36 hpf MCT8MO *vegfab* samples n = 2). Each dot represents a biological replicate; error bars represent standard deviation. For detailed statistics, see Supplementary Data 2.

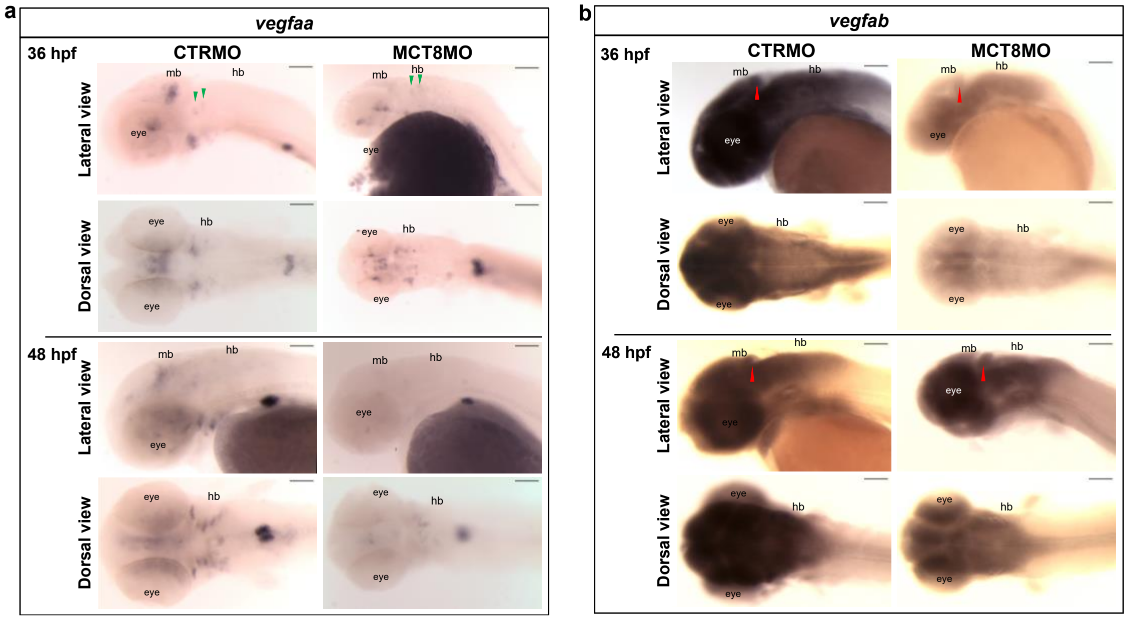

Supplementary Figure 3. MT3 regulates *vegfa* ligands expression in the zebrafish hindbrain. a) WISH expression analysis for *vegfaa* at 36 hpf and 48 hpf shows a clear decrease of expression in MCT8MO compared to CTRMO zebrafish embryos. Specific *vegfaa* signaling is indicated by green arrowheads of putative CtA location. **b)** WISH expression analysis for *vegfab* at 36 hpf and 48 hpf shows that the expression pattern was maintained between CTRMO and MCT8MO zebrafish embryos. The red arrowhead indicates the midbrain-hindbrain boundary (MHB). hb – hindbrain; mb – midbrain. A minimum of 10 zebrafish embryos per condition, time points and genes were analyzed. Scale bar: 100 μm.

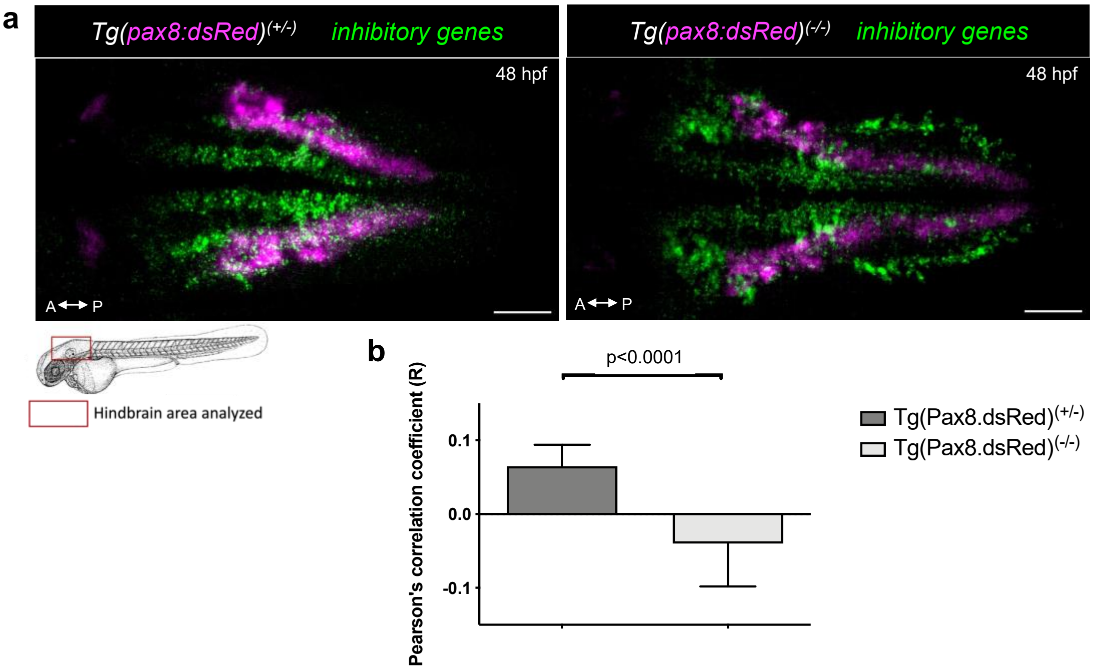

Supplementary Figure 4. Inhibition of *pax8* gene function modifies cell fate determination of the inhibitory interneurons. a) Dorsal view of fluorescent maximum projection images of the hindbrain of *Tg(pax8:DsRed)*$^{+/-}$ (heterozygote – control) and *Tg(pax8:DsRed)*$^{-/-}$ (homozygote – hypomorph mutant) zebrafish embryos at 48 hpf are represented. Embryos were submitted to double WISH for markers of DsRed (*pax8* lineage marker, magenta), glycinergic (glyt2A and glyt2B) and GABAergic (GAD67A and GAD76B) (labelled "inhibitory", probes were mixed, green). Scale bar: 50 μm. The red box indicates the hindbrain area analyzed by camera lucida drawings adapted from Kimmel. **b)** Image j plugin for Confined displacement algorithm (CDA) was used to analyze colocalization significance to compute the Pearson correlation coefficient between control *pax8*$^{+/-}$ (n = 14) and hypomorph *pax8*$^{-/-}$ mutant (n = 9) zebrafish embryos; Fisher's exact test; error bars represent standard deviation. For detailed statistics, see Supplementary Data 2.

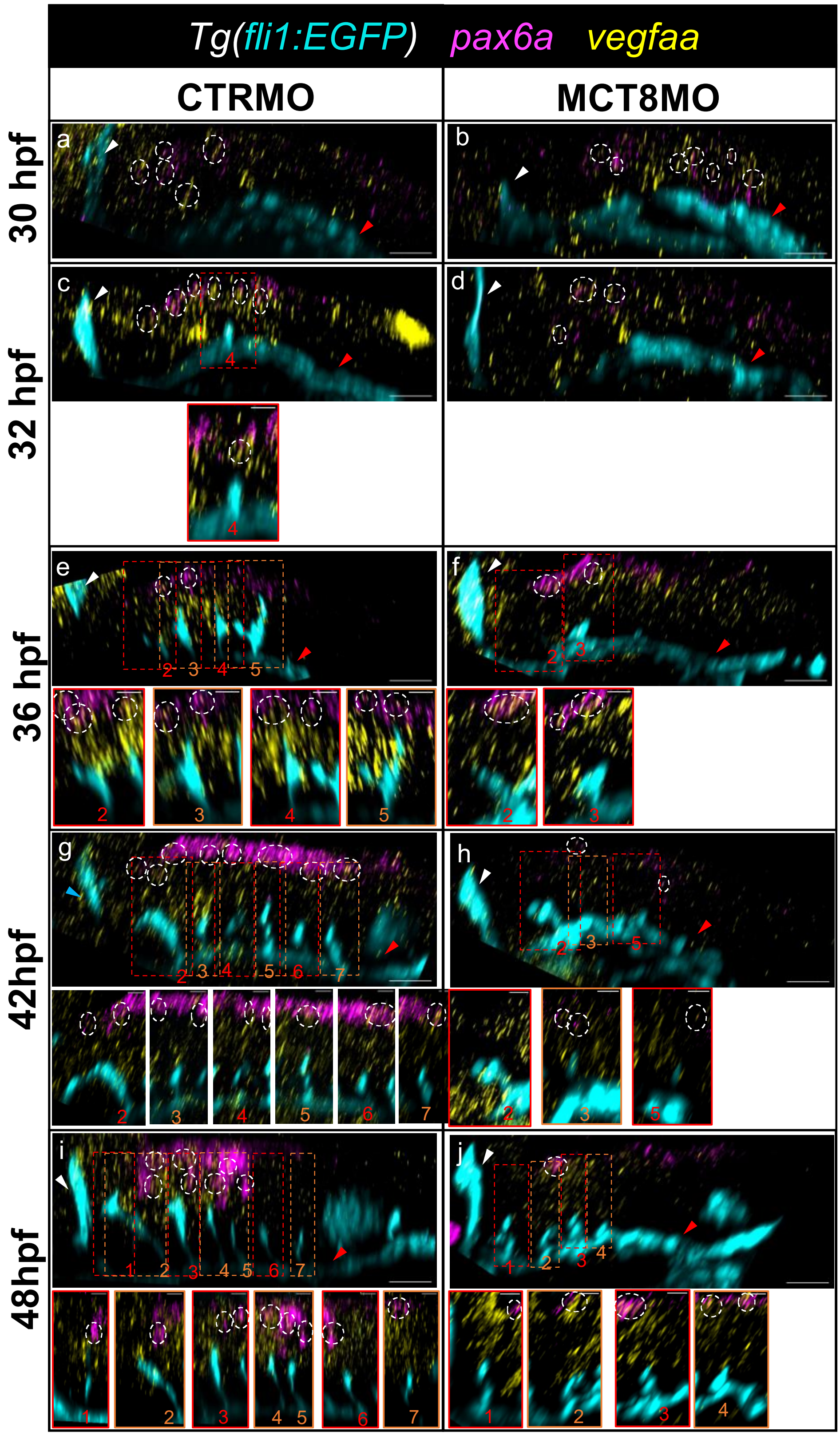

Supplementary Figure 5. *pax6a* and *vegfaa* expression colocalize during blood-hindbrain barrier development. Fluorescent maximum projection images of double WISH of *pax6a* (magenta) and *vegfaa* (yellow) and immunostained against GFP (endothelial marker, cyan) are represented for the

following stages and conditions: **(a)** 30 hpf CTRMO, **(b)** 30 hpf MCT8MO, **(c)** 32 hpf CTRMO, **(d)** 32 hpf MCT8MO, **(e)** 36 hpf CTRMO, **(f)** 36 hpf MCT8MO, **(g)** 42 hpf CTRMO, **(h)** 42 hpf MCT8MO, **(i)** 48 hpf CTRMO, and **(j)** 48 hpf MCT8MO. The hindbrain of CTRMO and MCT8MO zebrafish embryos were analyzed for colocalization of *pax6a* and *vegfaa* co-expressing cells (white dotted circles) during BHB development at different time points. Colocalization was determined by using the colormap colocalization plugin of Fiji software in the region of every CtA. Under each full hindbrain image, a maximum projection of the CtA, which is highlighted by a red or an orange dotted box, is represented to show the colocalization of *pax6a* and *vegfaa* expressing cells in the plane of the CtA migration. The white arrowhead represents the mid-cerebral vein (MCeV), and the red arrowhead represents the primordial hindbrain channels (PHBC). Red/orange numbers 1 – 7 indicate the CtA in its respective rhombomere. n = 8 (30 hpf CTRMO, 32 hpf MCT8MO), 9 (30 hpf MCT8MO, 36 hpf MCT8MO), 10 (all other stages and conditions). Scale bar: 50 μm and 10 μm.

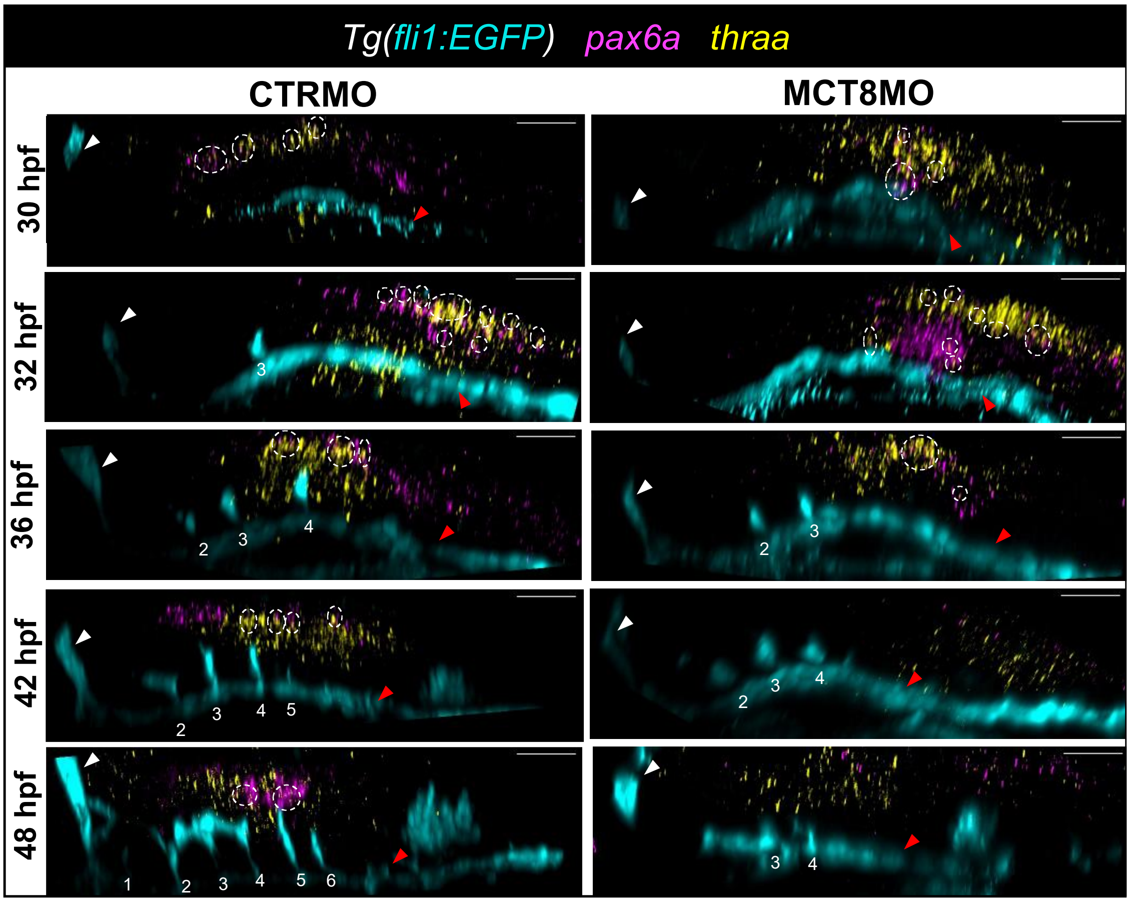

Supplementary Figure 6. *pax6a*-expressing cells colocalize with TH receptor *thraa*. Fluorescent maximum projection images of double WISH for *pax6a* (magenta) and *thraa* (yellow) and immunostained against GFP (endothelial marker, cyan) in CTRMO and MCT8MO zebrafish embryos at 30, 32, 36, 42 and 48 hpf are represented. The hindbrain of CTRMO and MCT8MO zebrafish embryos were analyzed for colocalization of *pax6a* and *thraa*-expressing cells (white dotted circles) during BHB development at different time points. Colocalization was determined by using the colormap colocalization plugin of Fiji software in the region of every CtA. The white arrowhead represents the mid-cerebral vein (MCeV), and the red arrowhead represents the primordial hindbrain channels (PHBC). Numbers 1 – 7 indicate the CtA in its respective rhombomere. Scale bar: 50 μm.

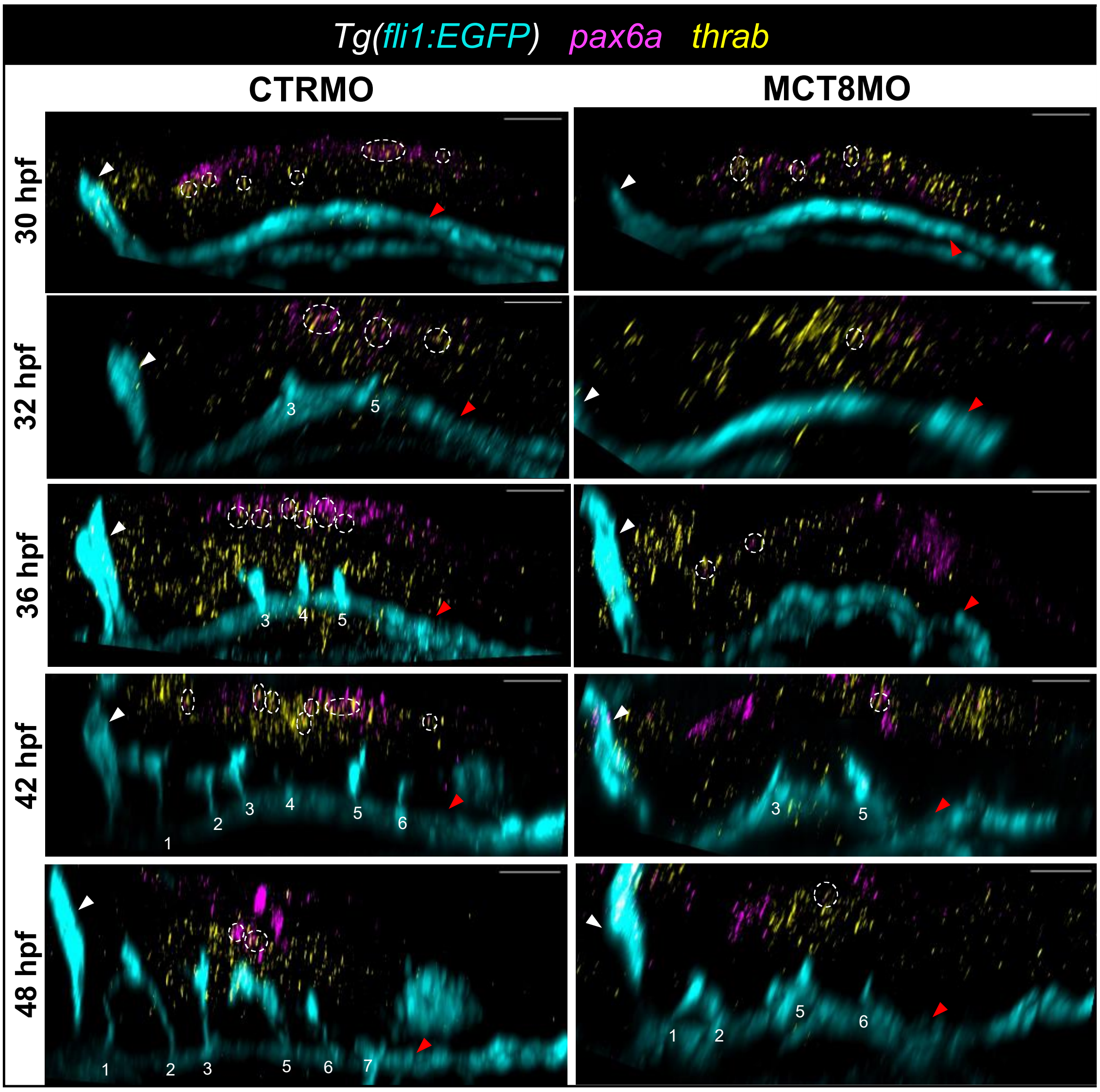

Supplementary Figure 7. *pax6a*-expressing cells colocalize with TH receptor *thrab*. Fluorescent maximum projection images of double WISH of *pax6a* (magenta) and *thrab* (yellow) and immunostained against GFP (endothelial marker, cyan) in CTRMO and MCT8MO zebrafish embryos at 30, 32, 36, 42 and 48 hpf are represented. The hindbrain of CTRMO and MCT8MO zebrafish embryos were analyzed for colocalization of *pax6a* and *thrab*-expressing cells (white dotted circles) during BHB development at different time points. Colocalization was determined by using the colormap colocalization plugin of Fiji software in the region of every CtA. The white arrowhead represents the mid-cerebral vein (MCeV), and the red arrowhead represents the primordial hindbrain channels (PHBC). Numbers 1 – 7 indicate the CtA in its respective rhombomere. Scale bar: 50 μm.

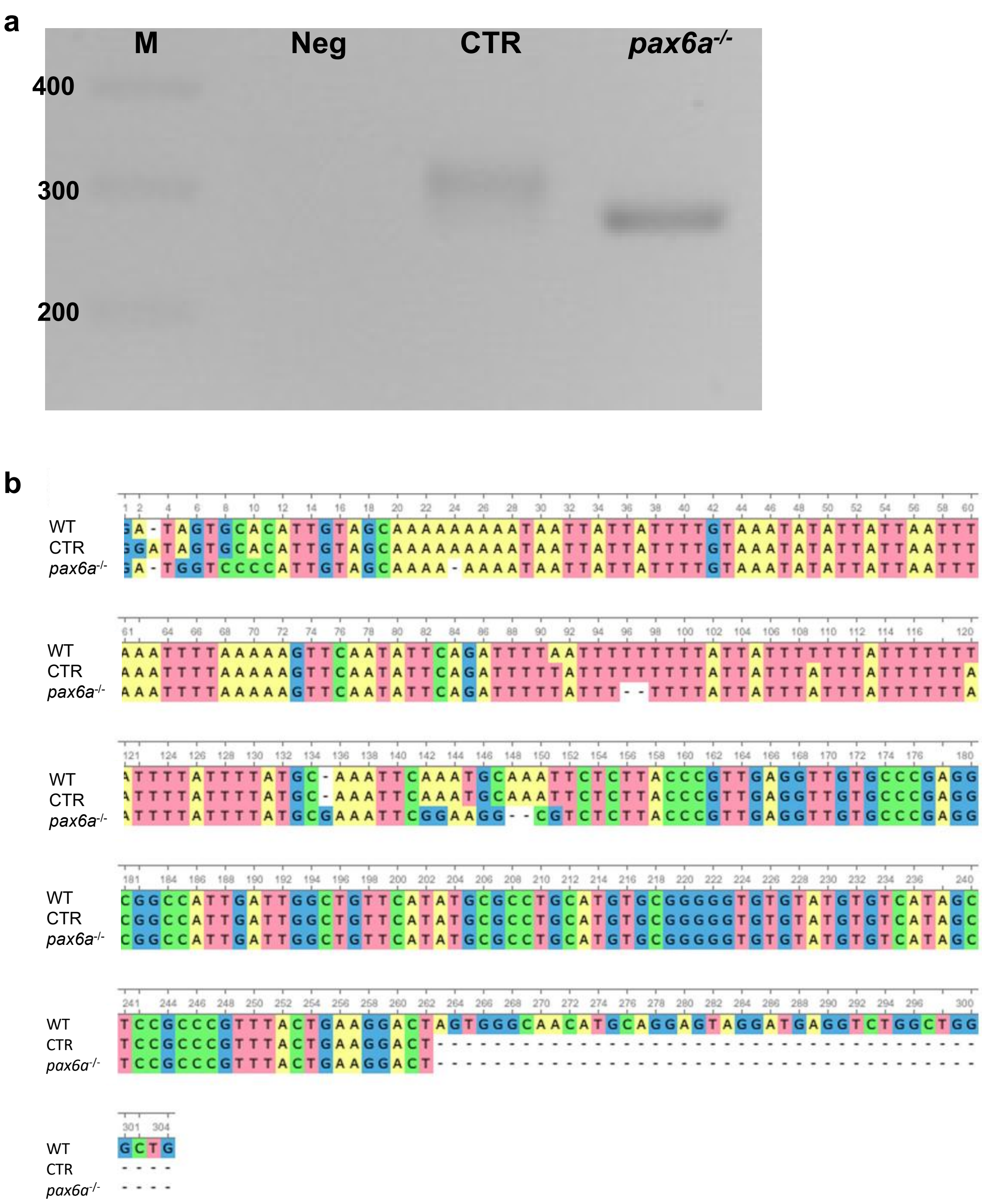

Supplementary Figure 8. CRISPR/Cas9 generated *pax6a* crispants gives rise to mutations in exon 7 that lead to the production of a truncated protein. a) PCR genotyping of not injected control embryos (CTR) and homozygotic *pax6a* mutant zebrafish embryos (*pax6a*$^{-/-}$). M – Gene marker in base pair; Neg – Negative PCR control. Uncropped gel is available in Supplementary Data 1. **b)** Sequence alignment of the canonical *pax6a* wildtype (WT) exon 7 genomic region, with sequenced not injected control embryo (CTR), and homozygotic *pax6a* mutant embryo (*pax6a*$^{-/-}$).
