# Supplementary material for: Maternal thyroid hormone is required to develop the hindbrain vasculature in zebrafish
Source: Commun Biol. 2025 Jul 1;8:960. doi: 10.1038/s42003-025-08404-1 (PMC12216513; doi:10.1038/s42003-025-08404-1)
Supplement: Supplementary file 3 — Description of Additional Supplementary Files [file 42003_2025_8404_MOESM3_ESM.pdf]

Description of Additional Supplementary Files

File name: Supplementary Data 1

Description: All source data used to produce the graphs

File name: Supplementary Data 2

Description: Detailed statistics
