# Supplementary material for: Maternal thyroid hormone is required to develop the hindbrain vasculature in zebrafish
Source: Commun Biol. 2025 Jul 1;8:960. doi: 10.1038/s42003-025-08404-1 (PMC12216513; doi:10.1038/s42003-025-08404-1)
Supplement: Supplementary file 5 — Supplementary data 2 [file 42003_2025_8404_MOESM5_ESM.pdf]

Supplementary Data 2 – Detailed statistics

**Figure**	**Statistics**	**Results**	**Post-hoc Test**	**Stage/ hindbrain structure/ gene analyzed**	**Comparison**	**Significance**	**P-value**
**1b**	Fisher's exact test (Two-sided)			32hpf	CtA1: CTRMO vs. MCT8MO	ns	>0.9999
					CtA2: CTRMO vs. MCT8MO	****	<0.0001
					CtA3: CTRMO vs. MCT8MO	****	<0.0001
					CtA4: CTRMO vs. MCT8MO	ns	0.5353
					CtA5: CTRMO vs. MCT8MO	**	0.0032
					CtA6: CTRMO vs. MCT8MO	ns	>0.9999
					CtA7: CTRMO vs. MCT8MO	ns	>0.9999
**1c**	Fisher's exact test (Two-sided)			36hpf	CtA1: CTRMO vs. MCT8MO	ns	0.1212
					CtA2: CTRMO vs. MCT8MO	****	<0.0001
					CtA3: CTRMO vs. MCT8MO	****	<0.0001
					CtA4: CTRMO vs. MCT8MO	****	<0.0001
					CtA5: CTRMO vs. MCT8MO	ns	0.4260
					CtA6: CTRMO vs. MCT8MO	ns	>0.9999
					CtA7: CTRMO vs. MCT8MO	ns	>0.9999
**1d**	Fisher's exact test (Two-sided)			48hpf	CtA1: CTRMO vs. MCT8MO	****	<0.0001
					CtA2: CTRMO vs. MCT8MO	****	<0.0001
					CtA3: CTRMO vs. MCT8MO	****	<0.0001
					CtA4: CTRMO vs. MCT8MO	****	<0.0001

					CtA5: CTRMO vs. MCT8MO	***	0.0005
					CtA6: CTRMO vs. MCT8MO	ns	0.5928
					CtA7: CTRMO vs. MCT8MO	ns	>0.9999
**1f**	One-way ANOVA	F(2, 45) = 34.28 P<0.0001	Bonferroni's multiple comparisons test	48hpf	CTRMO vs. MCT8MO	****	<0.0001
					CTRMO vs. MCT8MO + vegfaa mRNA	*	<0.05
					MCT8MO vs. MCT8MO + vegfaa mRNA	****	<0.0001
**1g**	Fisher's exact test (Two-sided)			48hpf	CtA1: CTRMO vs. MCT8MO	****	<0.0001
					CtA1: CTRMO vs. MCT8MO + vegfaa mRNA	ns	0.4725
					CtA1: MCT8MO vs. MCT8MO + vegfaa mRNA	****	<0.0001
					CtA2: CTRMO vs. MCT8MO	****	<0.0001
					CtA2: CTRMO vs. MCT8MO + vegfaa mRNA	ns	0.1941
					CtA2: MCT8MO vs. MCT8MO + vegfaa mRNA	****	<0.0001
					CtA3: CTRMO vs. MCT8MO	****	<0.0001
					CtA3: CTRMO vs. MCT8MO + vegfaa mRNA	ns	>0.9999
					CtA3: MCT8MO vs. MCT8MO + vegfaa mRNA	****	<0.0001
					CtA4: CTRMO vs. MCT8MO	****	<0.0001

				CtA4: CTRMO vs. MCT8MO + vegfaa mRNA	****	<0.0001
				CtA4: MCT8MO vs. MCT8MO + vegfaa mRNA	ns	0.1169
				CtA5: CTRMO vs. MCT8MO	****	<0.0001
				CtA5: CTRMO vs. MCT8MO + vegfaa mRNA	****	<0.0001
				CtA5: MCT8MO vs. MCT8MO + vegfaa mRNA	**	0.0070
				CtA6: CTRMO vs. MCT8MO	****	<0.0001
				CtA6: CTRMO vs. MCT8MO + vegfaa mRNA	****	<0.0001
				CtA6: MCT8MO vs. MCT8MO + vegfaa mRNA	***	0.0010
				CtA7: CTRMO vs. MCT8MO	****	<0.0001
				CtA7: CTRMO vs. MCT8MO + vegfaa mRNA	ns	0.6712
				CtA7: MCT8MO vs. MCT8MO + vegfaa mRNA	****	<0.0001
**2c**	unpaired t-test	t=0.06434, df=25 (Two-tailed)	PHBC	32 hpf: CTR vs. T3 treatment	ns	0.9492
		t=0.7549, df=32 (Two-tailed)		36 hpf: CTR vs. T3 treatment	ns	0.4558
		t=0.03913, df=41 (Two-tailed)		48 hpf: CTR vs. T3 treatment	ns	0.9690
**2d**	unpaired t-test	t=0.9971, df=22 (Two-tailed)	BA	32 hpf: CTR vs. T3 treatment	ns	0.3296
		t=0.02256, df=29 (Two-tailed)		36 hpf: CTR vs. T3 treatment	ns	0.9822

		t=0.3565, df=36 (Two-tailed)		48 hpf: CTR vs. T3 treatment	ns	0.7235
**2e**	unpaired t-test	t=0.9867, df=42 (Two-tailed)	CtAs	48 hpf: CTR vs. T3 treatment	ns	0.3295
**2f**	unpaired t-test	t=1.876, df=24 (Two-tailed)	PHBC	32 hpf: CTRMO vs. MCT8MO	ns	0.0729
		t=0.3890, df=27 (Two-tailed)		36 hpf: CTRMO vs. MCT8MO	ns	0.7004
		t=5.298, df=25 (Two-tailed)		48 hpf: CTRMO vs. MCT8MO	****	<0.0001
**2g**	unpaired t-test	t=1.232, df=20 (Two-tailed)	BA	32 hpf: CTRMO vs. MCT8MO	ns	0.2322
		t=4.651, df=25 (Two-tailed)		36 hpf: CTRMO vs. MCT8MO	****	<0.0001
		t=3.714, df=25 (Two-tailed)		48 hpf: CTRMO vs. MCT8MO	**	0.0010
**2h**	unpaired t-test	t=2.204, df=28 (Two-tailed)	CtAs	48 hpf: CTRMO vs. MCT8MO	*	0.0359
**3e**	unpaired t-test (Two-tailed)	Mann-Whitney test	48hpf	CtA1: *Tg(Pax8:dsRed)*+/- vs. *Tg(Pax8:dsRed)*-/-	ns	0.8407
				CtA2: *Tg(Pax8:dsRed)*+/- vs. *Tg(Pax8:dsRed)*-/-	ns	0.1329
				CtA3: *Tg(Pax8:dsRed)*+/- vs. *Tg(Pax8:dsRed)*-/-	ns	0.3772
				CtA4: *Tg(Pax8:dsRed)*+/- vs. *Tg(Pax8:dsRed)*-/-	ns	0.0542
				CtA5: *Tg(Pax8:dsRed)*+/- vs. *Tg(Pax8:dsRed)*-/-	ns	0.2416
				CtA6: *Tg(Pax8:dsRed)*+/- vs. *Tg(Pax8:dsRed)*-/-	ns	0.2581

				CtA7: *Tg(Pax8:dsRed)*+/- vs. *Tg(Pax8:dsRed)*-/-	**	0.0041
**3f**	unpaired t-test (Two-tailed)	Mann-Whitney test	48hpf	*Tg(Pax8:dsRed)*+/- vs. *Tg(Pax8:dsRed)*-/-	ns	0.6941
**4b**	unpaired t-test	t=1.374, df=15 (Two-tailed)	32hpf	CtA2: CTRMO vs. MCT8MO	ns	0.1895
		t=0.7519, df=15 (Two-tailed)		CtA3: CTRMO vs. MCT8MO	ns	0.4637
		t=2.129, df=15 (Two-tailed)		CtA4: CTRMO vs. MCT8MO	ns	0.0502
		t=0.8371, df=15 (Two-tailed)		CtA5: CTRMO vs. MCT8MO	ns	0.4157
		t=1.879, df=15 (Two-tailed)		CtA6: CTRMO vs. MCT8MO	ns	0.0799
		t=1.174, df=15 (Two-tailed)		CtA7: CTRMO vs. MCT8MO	ns	0.2586
**4c**	unpaired t-test	t=1.108, df=9 (Two-tailed)	36hpf	CtA1: CTRMO vs. MCT8MO	ns	0.2967
		t=1.883, df=9 (Two-tailed)		CtA2: CTRMO vs. MCT8MO	ns	0.924
		t=1.211, df=9 (Two-tailed)		CtA4: CTRMO vs. MCT8MO	ns	0.2568
		t=4.007, df=9 (Two-tailed)		CtA5: CTRMO vs. MCT8MO	**	0.0031
		t=0.5324, df=9 (Two-tailed)		CtA6: CTRMO vs. MCT8MO	ns	0.6074
		t=2.335, df=9 (Two-tailed)		CtA7: CTRMO vs. MCT8MO	*	0.0443
**4d**	unpaired t-test	t=1.080, df=15 (Two-tailed)	48hpf	CtA1: CTRMO vs. MCT8MO	ns	0.2972
		t=3.146, df=15 (Two-tailed)		CtA2: CTRMO vs. MCT8MO	**	0.0067
		t=1.891, df=15 (Two-tailed)		CtA3: CTRMO vs. MCT8MO	ns	0.0781
		t=3.007, df=15 (Two-tailed)		CtA4: CTRMO vs. MCT8MO	**	0.0089
		t=9.997, df=15 (Two-tailed)		CtA5: CTRMO vs. MCT8MO	****	<0.0001
		t=6.722, df=15 (Two-tailed)		CtA6: CTRMO vs. MCT8MO	****	<0.0001
		t=5.290, df=15 (Two-tailed)		CtA7: CTRMO vs. MCT8MO	****	<0.0001
**4e**	Fisher's exact test		48hpf	CtA2: CTRMO vs. MCT8MO	ns	>0.9999

	(Two-sided)		CtA3: CTRMO vs. MCT8MO	ns	>0.9999
			CtA4: CTRMO vs. MCT8MO	**	0.0050
			CtA5: CTRMO vs. MCT8MO	ns	0.5014
			CtA6: CTRMO vs. MCT8MO	****	<0.0001
			CtA7: CTRMO vs. MCT8MO	****	<0.0001
**5c**	Fisher's exact test (Two-sided)	CtA1	42hpf: CTRMO vs. MCT8MO	ns	>0.9999
			48hpf: CTRMO vs. MCT8MO	ns	0.3592
**5d**	Fisher's exact test (Two-sided)	CtA2	30hpf: CTRMO vs. MCT8MO	ns	>0.9999
			32hpf: CTRMO vs. MCT8MO	ns	>0.9999
			36hpf: CTRMO vs. MCT8MO	****	<0.0001
			42hpf: CTRMO vs. MCT8MO	***	0.0002
			48hpf: CTRMO vs. MCT8MO	***	0.0007
**5e**	Fisher's exact test (Two-sided)	CtA3	30hpf: CTRMO vs. MCT8MO	ns	>0.9999
			32hpf: CTRMO vs. MCT8MO	****	<0.0001
			36hpf: CTRMO vs. MCT8MO	ns	0.4751
			42hpf: CTRMO vs. MCT8MO	ns	0.3417
			48hpf: CTRMO vs. MCT8MO	ns	0.2081
**5f**	Fisher's exact test (Two-sided)	CtA4	30hpf: CTRMO vs. MCT8MO	ns	>0.9999
			32hpf: CTRMO vs. MCT8MO	****	<0.0001
			36hpf: CTRMO vs. MCT8MO	***	0.0006
			42hpf: CTRMO vs. MCT8MO	ns	>0.9999
			48hpf: CTRMO vs. MCT8MO	**	0.0063
**5g**	Fisher's exact test (Two-sided)	CtA5	30hpf: CTRMO vs. MCT8MO	ns	>0.9999
			32hpf: CTRMO vs. MCT8MO	****	<0.0001

					36hpf: CTRMO vs. MCT8MO	ns	0.7382
					42hpf: CTRMO vs. MCT8MO	*	0.0297
					48hpf: CTRMO vs. MCT8MO	ns	0.5053
**5h**	Fisher's exact test (Two-sided)			CtA6	30hpf: CTRMO vs. MCT8MO	ns	>0.9999
					32hpf: CTRMO vs. MCT8MO	ns	>0.9999
					36hpf: CTRMO vs. MCT8MO	****	<0.0001
					42hpf: CTRMO vs. MCT8MO	****	<0.0001
					48hpf: CTRMO vs. MCT8MO	ns	>0.9999
**5i**	Fisher's exact test (Two-sided)			CtA7	30hpf: CTRMO vs. MCT8MO	ns	>0.9999
					32hpf: CTRMO vs. MCT8MO	ns	>0.9999
					36hpf: CTRMO vs. MCT8MO	ns	>0.9999
					42hpf: CTRMO vs. MCT8MO	****	<0.0001
					48hpf: CTRMO vs. MCT8MO	ns	0.5944
**6a**	Two-way ANOVA	Row Factor: F (9, 23) = 0 P>0.9999 Column Factor: F (3, 23) = 0 P>0.9999	Tukey's multiple comparisons test	CtA1	32hpf: CTRMO_tip cell vs. CTRMO_vegfaa/pax6a cells		
					32hpf: CTRMO_tip cell vs. MCT8MO_tip cell		
					32hpf: CTRMO_vegfaa/pax6a cells vs. MCT8MO_vegfaa/pax6a cells		
					32hpf: MCT8MO_tip cell vs. MCT8MO_vegfaa/pax6a cell		
		Row Factor:	Tukey's multiple	CtA1	36hpf: CTRMO_tip cell vs. CTRMO_vegfaa/pax6a cells		

		F (9, 25) = 0,<br>P>0.9999<br>Column Factor:<br>F (3, 25) = 0,<br>P>0.9999	comparisons<br>test		36hpf: CTRMO_tip cell vs. MCT8MO_tip cell		
					36hpf: CTRMO_vegfaa/pax6a cells vs. MCT8MO_vegfaa/pax6a cells		
					36hpf: MCT8MO_tip cell vs. MCT8MO_vegfaa/pax6a cell		
		Row Factor:<br>F (9, 27) = 1.000<br>P=0.4635<br>Column Factor:<br>F (3, 27) = 2.250<br>P=0.1054	Tukey's multiple comparisons test	CtA1	42hpf: CTRMO_tip cell vs. CTRMO_vegfaa/pax6a cells	ns	$P_{adj} = 0.1720$
					42hpf: CTRMO_tip cell vs. MCT8MO_tip cell	ns	$P_{adj} = 0.1720$
					42 hpf: CTRMO_vegfaa/pax6a cells vs. MCT8MO_vegfaa/pax6a cells	ns	$P_{adj} > 0.9999$
					42hpf: MCT8MO_tip cell vs. MCT8MO_vegfaa/pax6a cell	ns	$P_{adj} > 0.9999$
		Row Factor:<br>F (9, 27) = 0.9013<br>P=0.5378<br>Column Factor:<br>F (3, 27) = 11.00<br>P<0.0001	Tukey's multiple comparisons test	CtA1	48hpf: CTRMO_tip cell vs. CTRMO_vegfaa/pax6a cells	**	$P_{adj} = 0.0066$
					48hpf: CTRMO_tip cell vs. MCT8MO_tip cell	***	$P_{adj} = 0.0002$
					48hpf: CTRMO_vegfaa/pax6a cells vs. MCT8MO_vegfaa/pax6a cells	ns	$P_{adj} = 0.4832$
					48hpf: MCT8MO_tip cell vs. MCT8MO_vegfaa/pax6a cell	ns	$P_{adj} = 0.9986$
**6b**	Two-way ANOVA	Row Factor:	Tukey's multiple	CtA2	32hpf: CTRMO_tip cell vs. CTRMO_vegfaa/pax6a cells		

		F (9, 23) = 0<br>P>0.9999<br>Column Factor:<br>F (3, 23) = 0<br>P>0.9999	comparisons test		32hpf: CTRMO_tip cell vs. MCT8MO_tip cell		
					32hpf: CTRMO_vegfaa/pax6a cells vs. MCT8MO_vegfaa/pax6a cells		
					32hpf: MCT8MO_tip cell vs. MCT8MO_vegfaa/pax6a cell		
		Row Factor:<br>F (9, 25) = 2.411<br>P=9.0398<br>Column Factor:<br>F (3, 25) = 3.090<br>P=0.0453	Tukey's multiple comparisons test	CtA2	36hpf: CTRMO_tip cell vs. CTRMO_vegfaa/pax6a cells	ns	P_{adj} = 0.9478
					36hpf: CTRMO_tip cell vs. MCT8MO_tip cell	ns	P_{adj} = 0.6176
					36hpf: CTRMO_vegfaa/pax6a cells vs. MCT8MO_vegfaa/pax6a cells	ns	P_{adj} = 0.5782
					36hpf: MCT8MO_tip cell vs. MCT8MO_vegfaa/pax6a cell	*	P_{adj} = 0.0291
		Row Factor:<br>F (9, 27) = 2.137<br>P=0.0616<br>Column Factor:<br>F (3, 27) = 10.38<br>P=0.0001	Tukey's multiple comparisons test	CtA2	42hpf: CTRMO_tip cell vs. CTRMO_vegfaa/pax6a cells	**	P_{adj} = 0.0012
					42hpf: CTRMO_tip cell vs. MCT8MO_tip cell	**	P_{adj} = 0.0093
					42 hpf: CTRMO_vegfaa/pax6a cells vs. MCT8MO_vegfaa/pax6a cells	ns	P_{adj} = 0.7647
					42hpf: MCT8MO_tip cell vs. MCT8MO_vegfaa/pax6a cell	ns	P_{adj} = 0.3046
		Row Factor:	Tukey's multiple	CtA2	48hpf: CTRMO_tip cell vs. CTRMO_vegfaa/pax6a cells	**	P_{adj} = 0.0023

		F (9, 27) = 3.918<br>P=0.0028<br>Column Factor:<br>F (3, 27) = 15,02<br>P<0.0001	comparisons<br>test		48hpf: CTRMO_tip cell vs. MCT8MO_tip cell	***	P_{adj} = 0.0001
					48hpf: CTRMO_vegfaa/pax6a cells vs. MCT8MO_vegfaa/pax6a cells	ns	P_{adj} = 0.1268
					48hpf: MCT8MO_tip cell vs. MCT8MO_vegfaa/pax6a cell	ns	P_{adj} = 0.6854
**6c**	Two-way ANOVA	Row Factor:<br>F (9, 23) = 2.209<br>P=0.0605<br>Column Factor:<br>F (3, 23) = 10.63<br>P=0.0001	Tukey's multiple comparisons test	CtA3	32hpf: CTRMO_tip cell vs. CTRMO_vegfaa/pax6a cells	ns	P_{adj} = 0.6270
					32hpf: CTRMO_tip cell vs. MCT8MO_tip cell	***	P_{adj} = 0.0008
					32hpf: CTRMO_vegfaa/pax6a cells vs. MCT8MO_vegfaa/pax6a cells	*	P_{adj} = 0.0110
					32hpf: MCT8MO_tip cell vs. MCT8MO_vegfaa/pax6a cell	ns	P_{adj} > 0.9999
		Row Factor:<br>F (9, 25) = 2.964<br>P=0.0153<br>Column Factor:<br>F (3, 25) = 5.446<br>P=0.0051	Tukey's multiple comparisons test	CtA3	36hpf: CTRMO_tip cell vs. CTRMO_vegfaa/pax6a cells	ns	P_{adj} = 0.5885
					36hpf: CTRMO_tip cell vs. MCT8MO_tip cell	*	P_{adj} = 0.0230
					36hpf: CTRMO_vegfaa/pax6a cells vs. MCT8MO_vegfaa/pax6a cells	ns	P_{adj} = 0.1128
					36hpf: MCT8MO_tip cell vs. MCT8MO_vegfaa/pax6a cell	ns	P_{adj} = 0.9649
		Row Factor:	Tukey's multiple	CtA3	42hpf: CTRMO_tip cell vs. CTRMO_vegfaa/pax6a cells	*	P_{adj} = 0.0359

		F (9, 27) = 3.424 P=0.0063 Column Factor: F (3, 27) = 6.659 P=0.0016	comparisons test		42hpf: CTRMO_tip cell vs. MCT8MO_tip cell	ns	P_{adj} = 0.0830
					42 hpf: CTRMO_vegfaa/pax6a cells vs. MCT8MO_vegfaa/pax6a cells	ns	P_{adj} = 0.4445
					42hpf: MCT8MO_tip cell vs. MCT8MO_vegfaa/pax6a cell	ns	P_{adj} = 0.2518
		Row Factor: F (9, 27) = 0.6706, P=0.7279 Column Factor: F (3, 27) = 3.052, P=0.0455	Tukey's multiple comparisons test	CtA3	48hpf: CTRMO_tip cell vs. CTRMO_vegfaa/pax6a cells	*	P_{adj} = 0.0384
					48hpf: CTRMO_tip cell vs. MCT8MO_tip cell	ns	P_{adj} = 0.6911
					48hpf: CTRMO_vegfaa/pax6a cells vs. MCT8MO_vegfaa/pax6a cells	ns	P_{adj} = 0.8632
					48hpf: MCT8MO_tip cell vs. MCT8MO_vegfaa/pax6a cell	ns	P_{adj} = 0.7652
**6d**	Two-way ANOVA	Row Factor: F (9, 23) = 3.115 P=0.0134 Column Factor: F (3, 23) = 4.333 P=0.0147	Tukey's multiple comparisons test	CtA4	32hpf: CTRMO_tip cell vs. CTRMO_vegfaa/pax6a cells	ns	P_{adj} = 0.9947
					32hpf: CTRMO_tip cell vs. MCT8MO_tip cell	ns	P_{adj} = 0.0869
					32hpf: CTRMO_vegfaa/pax6a cells vs. MCT8MO_vegfaa/pax6a cells	ns	P_{adj} = 0.0550
					32hpf: MCT8MO_tip cell vs. MCT8MO_vegfaa/pax6a cell	ns	P_{adj} > 0.9999
		Row Factor: F (9, 25) = 1.963	Tukey's multiple	CtA4	36hpf: CTRMO_tip cell vs. CTRMO_vegfaa/pax6a cells	ns	P_{adj} = 0.9970

		P=0.0885<br>Column Factor:<br>F (3, 25) = 9.647<br>P=0.0002	comparisons<br>test		36hpf: CTRMO_tip cell vs. MCT8MO_tip cell	**	P_{adj} = 0.0030
					36hpf: CTRMO_vegfaa/pax6a cells vs. MCT8MO_vegfaa/pax6a cells	**	P_{adj} = 0.0048
					36hpf: MCT8MO_tip cell vs. MCT8MO_vegfaa/pax6a cell	ns	P_{adj} > 0.9999
		Row Factor:<br>F (9, 27) = 2.490<br>P=0.0322<br>Column Factor:<br>F (3, 27) = 8.550<br>P=0.0004	Tukey's multiple comparisons test	CtA4	42hpf: CTRMO_tip cell vs. CTRMO_vegfaa/pax6a cells	ns	P_{adj} = 0.5859
					42hpf: CTRMO_tip cell vs. MCT8MO_tip cell	**	P_{adj} = 0.0020
					42 hpf: CTRMO_vegfaa/pax6a cells vs. MCT8MO_vegfaa/pax6a cells	*	P_{adj} = 0.0379
					42hpf: MCT8MO_tip cell vs. MCT8MO_vegfaa/pax6a cell	ns	P_{adj} = 0.9999
		Row Factor:<br>F (9, 27) = 4.861<br>P=0.0006<br>Column Factor:<br>F (3, 27) = 11.80<br>P<0.0001	Tukey's multiple comparisons test	CtA4	48hpf: CTRMO_tip cell vs. CTRMO_vegfaa/pax6a cells	ns	P_{adj} = 0.9860
					48hpf: CTRMO_tip cell vs. MCT8MO_tip cell	**	P_{adj} = 0.0024
					48hpf: CTRMO_vegfaa/pax6a cells vs. MCT8MO_vegfaa/pax6a cells	***	P_{adj} = 0.0009
					48hpf: MCT8MO_tip cell vs. MCT8MO_vegfaa/pax6a cell	ns	P_{adj} = 0.8957
**6e**	Two-way ANOVA	Row Factor:	Tukey's multiple	CtA5	32hpf: CTRMO_tip cell vs. CTRMO_vegfaa/pax6a cells	ns	P_{adj} = 0.2870

		F (9, 23) = 1.480 P=0.2139 Column Factor: F (3, 23) = 3.764 P=0.0247	comparisons test		32hpf: CTRMO_tip cell vs. MCT8MO_tip cell	*	$P_{adj} = 0.0389$
					32hpf: CTRMO_vegfaa/pax6a cells vs. MCT8MO_vegfaa/pax6a cells	ns	$P_{adj} = 0.6280$
					32hpf: MCT8MO_tip cell vs. MCT8MO_vegfaa/pax6a cell	ns	$P_{adj} > 0.9999$
		Row Factor: F (9, 25) = 6.693 P<0.0001 Column Factor: F (3, 25) = 19.28 P<0.0001	Tukey's multiple comparisons test	CtA5	36hpf: CTRMO_tip cell vs. CTRMO_vegfaa/pax6a cells	ns	$P_{adj} = 0.7657$
					36hpf: CTRMO_tip cell vs. MCT8MO_tip cell	****	$P_{adj} < 0.0001$
					36hpf: CTRMO_vegfaa/pax6a cells vs. MCT8MO_vegfaa/pax6a cells	***	$P_{adj} = 0.0002$
					36hpf: MCT8MO_tip cell vs. MCT8MO_vegfaa/pax6a cell	ns	$P_{adj} > 0.9999$
		Row Factor: F (9, 27) = 2.253 P=0.0497 Column Factor: F (3, 27) = 19.17 P<0.0001	Tukey's multiple comparisons test	CtA5	42hpf: CTRMO_tip cell vs. CTRMO_vegfaa/pax6a cells	ns	$P_{adj} = 0.5910$
					42hpf: CTRMO_tip cell vs. MCT8MO_tip cell	****	$P_{adj} < 0.0001$
					42 hpf: CTRMO_vegfaa/pax6a cells vs. MCT8MO_vegfaa/pax6a cells	***	$P_{adj} = 0.0003$
					42hpf: MCT8MO_tip cell vs. MCT8MO_vegfaa/pax6a cell	ns	$P_{adj} = 0.9955$
		Row Factor:	Tukey's multiple	CtA5	48hpf: CTRMO_tip cell vs. CTRMO_vegfaa/pax6a cells	ns	$P_{adj} = 0.3005$

		F (9, 27) = 3.072<br>P=0.0114<br>Column Factor:<br>F (3, 27) = 1.859<br>P=0.1605	comparisons test		48hpf: CTRMO_tip cell vs. MCT8MO_tip cell	ns	$P_{adj} = 0.9991$
					48hpf: CTRMO_vegfaa/pax6a cells vs. MCT8MO_vegfaa/pax6a cells	ns	$P_{adj} = 0.9995$
					48hpf: MCT8MO_tip cell vs. MCT8MO_vegfaa/pax6a cell	ns	$P_{adj} = 0.4279$
**6f**	Two-way ANOVA	Row Factor:<br>F (9, 23) = 0<br>P>0.9999<br>Column Factor:<br>F (3, 23) = 0<br>P>0.9999	Tukey's multiple comparisons test	CtA6	32hpf: CTRMO_tip cell vs. CTRMO_vegfaa/pax6a cells		
					32hpf: CTRMO_tip cell vs. MCT8MO_tip cell		
					32hpf: CTRMO_vegfaa/pax6a cells vs. MCT8MO_vegfaa/pax6a cells		
					32hpf: MCT8MO_tip cell vs. MCT8MO_vegfaa/pax6a cell		
		Row Factor:<br>F (9, 25) = 2.922<br>P=0.0164<br>Column Factor:<br>F (3, 25) = 2.316<br>P=0.1001	Tukey's multiple comparisons test	CtA6	36hpf: CTRMO_tip cell vs. CTRMO_vegfaa/pax6a cells	ns	$P_{adj} = 0.9999$
					36hpf: CTRMO_tip cell vs. MCT8MO_tip cell	ns	$P_{adj} = 0.2451$
					36hpf: CTRMO_vegfaa/pax6a cells vs. MCT8MO_vegfaa/pax6a cells	ns	$P_{adj} = 0.2732$
					36hpf: MCT8MO_tip cell vs. MCT8MO_vegfaa/pax6a cell	ns	$P_{adj} > 0.9999$
			Tukey's multiple	CtA6	42hpf: CTRMO_tip cell vs. CTRMO_vegfaa/pax6a cells	ns	$P_{adj} = 0.8910$

		Row Factor: F (9, 27) = 1.933, P=0.0898 Column Factor: F (3, 27) = 9.959, P=0.0001	comparisons test		42hpf: CTRMO_tip cell vs. MCT8MO_tip cell	**	$P_{adj} = 0.0019$
					42 hpf: CTRMO_vegfaa/pax6a cells vs. MCT8MO_vegfaa/pax6a cells	**	$P_{adj} = 0.0068$
					42hpf: MCT8MO_tip cell vs. MCT8MO_vegfaa/pax6a cell	ns	$P_{adj} = 0.9959$
		Row Factor: F (9, 27) = 1.221 P=0.3235 Column Factor: F (3, 27) = 22.50 P<0.0001	Tukey's multiple comparisons test	CtA6	48hpf: CTRMO_tip cell vs. CTRMO_vegfaa/pax6a cells	****	$P_{adj} < 0.0001$
					48hpf: CTRMO_tip cell vs. MCT8MO_tip cell	****	$P_{adj} < 0.0001$
					48hpf: CTRMO_vegfaa/pax6a cells vs. MCT8MO_vegfaa/pax6a cells	ns	$P_{adj} = 0.8505$
					48hpf: MCT8MO_tip cell vs. MCT8MO_vegfaa/pax6a cell	ns	$P_{adj} > 0.9999$
**6g**	Two-way ANOVA	Row Factor: F (9, 23) = 0 P>0.9999 Column Factor: F (3, 23) = 0 P>0.9999	Tukey's multiple comparisons test	CtA7	32hpf: CTRMO_tip cell vs. CTRMO_vegfaa/pax6a cells		
					32hpf: CTRMO_tip cell vs. MCT8MO_tip cell		
					32hpf: CTRMO_vegfaa/pax6a cells vs. MCT8MO_vegfaa/pax6a cells		
					32hpf: MCT8MO_tip cell vs. MCT8MO_vegfaa/pax6a cell		
		Row Factor:	Tukey's multiple	CtA7	36hpf: CTRMO_tip cell vs. CTRMO_vegfaa/pax6a cells		

		F (9, 25) = 0<br>P>0.9999<br>Column Factor:<br>F (3, 25) = 0<br>P>0.9999	comparisons<br>test		36hpf: CTRMO_tip cell vs. MCT8MO_tip cell		
					36hpf: CTRMO_vegfaa/pax6a cells vs. MCT8MO_vegfaa/pax6a cells		
					36hpf: MCT8MO_tip cell vs. MCT8MO_vegfaa/pax6a cell		
		Row Factor:<br>F (9, 27) = 2.693<br>P=0.0224<br>Column Factor:<br>F (3, 27) = 5.370<br>P=0.0050	Tukey's multiple comparisons test	CtA7	42hpf: CTRMO_tip cell vs. CTRMO_vegfaa/pax6a cells	ns	$P_{adj} = 0.8054$
					42hpf: CTRMO_tip cell vs. MCT8MO_tip cell	*	$P_{adj} = 0.0167$
					42 hpf: CTRMO_vegfaa/pax6a cells vs. MCT8MO_vegfaa/pax6a cells	ns	$P_{adj} = 0.1192$
					42hpf: MCT8MO_tip cell vs. MCT8MO_vegfaa/pax6a cell	ns	$P_{adj} > 0.9999$
		Row Factor:<br>F (9, 27) = 1.988<br>P=0.0810<br>Column Factor:<br>F (3, 27) = 19.81<br>P<0.0001	Tukey's multiple comparisons test	CtA7	48hpf: CTRMO_tip cell vs. CTRMO_vegfaa/pax6a cells	****	$P_{adj} < 0.0001$
					48hpf: CTRMO_tip cell vs. MCT8MO_tip cell	****	$P_{adj} < 0.0001$
					48hpf: CTRMO_vegfaa/pax6a cells vs. MCT8MO_vegfaa/pax6a cells	ns	$P_{adj} = 0.8787$
					48hpf: MCT8MO_tip cell vs. MCT8MO_vegfaa/pax6a cell	ns	$P_{adj} = 0.8650$
**7b**	Fisher's exact test<br>(Two-sided)			CtA1	48hpf: CTRMO vs. MCT8MO	ns	>0.9999

**7c**	Fisher's exact test (Two-sided)	CtA2	36hpf: CTRMO vs. MCT8MO	ns	>0.9999
			42hpf: CTRMO vs. MCT8MO	ns	>0.9999
			48hpf: CTRMO vs. MCT8MO	ns	>0.9999
**7d**	Fisher's exact test (Two-sided)	CtA3	32hpf: CTRMO vs. MCT8MO	ns	>0.9999
			36hpf: CTRMO vs. MCT8MO	*	0.0249
			42hpf: CTRMO vs. MCT8MO	****	<0.0001
			48hpf: CTRMO vs. MCT8MO	ns	>0.9999
**7e**	Fisher's exact test (Two-sided)	CtA4	32hpf: CTRMO vs. MCT8MO	ns	>0.9999
			36hpf: CTRMO vs. MCT8MO	ns	>0.9999
			42hpf: CTRMO vs. MCT8MO	****	<0.0001
			48hpf: CTRMO vs. MCT8MO	****	<0.0001
**7f**	Fisher's exact test (Two-sided)	CtA5	30hpf: CTRMO vs. MCT8MO	ns	>0.9999
			32hpf: CTRMO vs. MCT8MO	ns	>0.9999
			36hpf: CTRMO vs. MCT8MO	ns	>0.9999
			42hpf: CTRMO vs. MCT8MO	****	<0.0001
			48hpf: CTRMO vs. MCT8MO	****	<0.0001
**7g**	Fisher's exact test (Two-sided)	CtA6	30hpf: CTRMO vs. MCT8MO	ns	>0.9999
			32hpf: CTRMO vs. MCT8MO	ns	>0.9999
			36hpf: CTRMO vs. MCT8MO	ns	>0.9999
			42hpf: CTRMO vs. MCT8MO	ns	>0.9999
			48hpf: CTRMO vs. MCT8MO	ns	>0.9999
**7h**	Fisher's exact test (Two-sided)	CtA7	30hpf: CTRMO vs. MCT8MO	ns	>0.9999
			32hpf: CTRMO vs. MCT8MO	ns	>0.9999
			36hpf: CTRMO vs. MCT8MO	ns	>0.9999

			42hpf: CTRMO vs. MCT8MO	ns	>0.9999
			48hpf: CTRMO vs. MCT8MO	ns	>0.9999
**8a**	Fisher's exact test (Two-sided)	CtA1	42hpf: CTRMO vs. MCT8MO	ns	>0.9999
			48hpf: CTRMO vs. MCT8MO	ns	>0.9999
**8b**	Fisher's exact test (Two-sided)	CtA2	36hpf: CTRMO vs. MCT8MO	ns	>0.9999
			42hpf: CTRMO vs. MCT8MO	ns	>0.9999
			48hpf: CTRMO vs. MCT8MO	ns	>0.9999
**8c**	Fisher's exact test (Two-sided)	CtA3	30hpf: CTRMO vs. MCT8MO	ns	>0.9999
			32hpf: CTRMO vs. MCT8MO	ns	>0.9999
			36hpf: CTRMO vs. MCT8MO	**	0.0041
			42hpf: CTRMO vs. MCT8MO	ns	>0.9999
			48hpf: CTRMO vs. MCT8MO	****	<0.0001
**8d**	Fisher's exact test (Two-sided)	CtA4	32hpf: CTRMO vs. MCT8MO	ns	>0.9999
			36hpf: CTRMO vs. MCT8MO	*	0.0105
			42hpf: CTRMO vs. MCT8MO	ns	>0.9999
			48hpf: CTRMO vs. MCT8MO	ns	>0.9999
**8e**	Fisher's exact test (Two-sided)	CtA5	30hpf: CTRMO vs. MCT8MO	ns	>0.9999
			32hpf: CTRMO vs. MCT8MO	ns	>0.9999
			36hpf: CTRMO vs. MCT8MO	****	<0.0001
			42hpf: CTRMO vs. MCT8MO	ns	>0.9999
			48hpf: CTRMO vs. MCT8MO	ns	>0.9999
**8f**	Fisher's exact test (Two-sided)	CtA6	30hpf: CTRMO vs. MCT8MO	ns	>0.9999
			32hpf: CTRMO vs. MCT8MO	ns	>0.9999
			36hpf: CTRMO vs. MCT8MO	ns	>0.9999

			42hpf: CTRMO vs. MCT8MO	ns	>0.9999
			48hpf: CTRMO vs. MCT8MO	ns	>0.9999
**8g**	Fisher's exact test (Two-sided)	CtA7	30hpf: CTRMO vs. MCT8MO	ns	>0.9999
			32hpf: CTRMO vs. MCT8MO	ns	>0.9999
			36hpf: CTRMO vs. MCT8MO	ns	>0.9999
			48hpf: CTRMO vs. MCT8MO	ns	>0.9999
**8h**	Fisher's exact test (Two-sided)	CtA1	30hpf: CTRMO vs. MCT8MO	ns	>0.9999
			32hpf: CTRMO vs. MCT8MO	ns	>0.9999
			36hpf: CTRMO vs. MCT8MO	ns	>0.9999
			42hpf: CTRMO vs. MCT8MO	ns	>0.9999
			48hpf: CTRMO vs. MCT8MO	ns	>0.9999
**8i**	Fisher's exact test (Two-sided)	CtA2	32hpf: CTRMO vs. MCT8MO	ns	>0.9999
			36hpf: CTRMO vs. MCT8MO	ns	>0.9999
			42hpf: CTRMO vs. MCT8MO	****	<0.0001
			48hpf: CTRMO vs. MCT8MO	****	<0.0001
**8j**	Fisher's exact test (Two-sided)	CtA3	30hpf: CTRMO vs. MCT8MO	ns	>0.9999
			32hpf: CTRMO vs. MCT8MO	ns	>0.9999
			36hpf: CTRMO vs. MCT8MO	****	<0.0001
			42hpf: CTRMO vs. MCT8MO	ns	0.1733
			48hpf: CTRMO vs. MCT8MO	ns	>0.9999
**8k**	Fisher's exact test (Two-sided)	CtA4	30hpf: CTRMO vs. MCT8MO	ns	>0.9999
			32hpf: CTRMO vs. MCT8MO	***	0.0001
			36hpf: CTRMO vs. MCT8MO	****	<0.0001
			42hpf: CTRMO vs. MCT8MO	****	<0.0001

				48hpf: CTRMO vs. MCT8MO	**	0.0089
**8l**	Fisher's exact test (Two-sided)		CtA5	30hpf: CTRMO vs. MCT8MO	ns	>0.9999
				32hpf: CTRMO vs. MCT8MO	****	<0.0001
				36hpf: CTRMO vs. MCT8MO	****	<0.0001
				42hpf: CTRMO vs. MCT8MO	****	<0.0001
				48hpf: CTRMO vs. MCT8MO	***	0.0002
**8m**	Fisher's exact test (Two-sided)		CtA6	30hpf: CTRMO vs. MCT8MO	ns	>0.9999
				32hpf: CTRMO vs. MCT8MO	ns	>0.9999
				36hpf: CTRMO vs. MCT8MO	ns	>0.9999
				42hpf: CTRMO vs. MCT8MO	*	0.0105
				48hpf: CTRMO vs. MCT8MO	***	0.0002
**8n**	Fisher's exact test (Two-sided)		CtA7	42hpf: CTRMO vs. MCT8MO	ns	>0.9999
				48hpf: CTRMO vs. MCT8MO	ns	>0.9999
**Supplementary Figure 2c**	Unpaired t-test	t=0.6616, df=8 (Two-tailed)	*vegfaa*	28hpf: CTRMO vs. MCT8MO	ns	0.5268
		t=0.7170, df=8 (Two-tailed)		30hpf: CTRMO vs. MCT8MO	ns	0.4938
		t=2.115, df=8 (Two-tailed)		32hpf: CTRMO vs. MCT8MO	ns	0.0674
		t=0.6299, df=8 (Two-tailed)		36hpf: CTRMO vs. MCT8MO	ns	0.5463
		t=0.8535, df=8 (Two-tailed)		44hpf: CTRMO vs. MCT8MO	ns	0.4182
		t=0.02860, df=8 (Two-tailed)		48hpf: CTRMO vs. MCT8MO	ns	0.9779
		t=1.901, df=8 (Two-tailed)		54hpf: CTRMO vs. MCT8MO	ns	0.0938
		t=0.4081, df=8 (Two-tailed)		72hpf: CTRMO vs. MCT8MO	ns	0.6939
**Supplementary Figure 2d**	Unpaired t-test	t=1.380, df=8 (Two-tailed)	*vegfab*	28hpf: CTRMO vs. MCT8MO	ns	0.2051
		t=0.8391, df=8 (Two-tailed)		30hpf: CTRMO vs. MCT8MO	ns	0.4258
		t=0.2008, df=8 (Two-tailed)		32hpf: CTRMO vs. MCT8MO	ns	0.8459

		t=0.1715, df=5 (Two-tailed)		36hpf: CTRMO vs. MCT8MO	ns	0.8707
		t=2.106, df=8 (Two-tailed)		44hpf: CTRMO vs. MCT8MO	ns	0.0683
		t=1.472, df=8 (Two-tailed)		48hpf: CTRMO vs. MCT8MO	ns	0.1793
		t=2.626, df=8 (Two-tailed)		54hpf: CTRMO vs. MCT8MO	*	0.0304
		t=1.319, df=8 (Two-tailed)		72hpf: CTRMO vs. MCT8MO	ns	0.2237
**Supplementary Figure 2e**	Unpaired t-test	t=1.697, df=8 (Two-tailed)	*notch1b*	28hpf: CTRMO vs. MCT8MO	ns	0.1281
		t=0.04929, df=8 (Two-tailed)		30hpf: CTRMO vs. MCT8MO	ns	0.9619
		t=0.2795, df=8 (Two-tailed)		32hpf: CTRMO vs. MCT8MO	ns	0.7869
		t=1462, df=8 (Two-tailed)		36hpf: CTRMO vs. MCT8MO	ns	0.8874
		t=0.1716, df=8 (Two-tailed)		44hpf: CTRMO vs. MCT8MO	ns	0.8680
		t=0.09040, df=8 (Two-tailed)		48hpf: CTRMO vs. MCT8MO	ns	0.9302
		t=0.8844, df=8 (Two-tailed)		54hpf: CTRMO vs. MCT8MO	ns	0.4023
		t=0.2142, df=8 (Two-tailed)		72hpf: CTRMO vs. MCT8MO	ns	0.8358
**Supplementary Figure 2f**	Unpaired t-test	t=0.1066, df=8 (Two-tailed)	*nrp1a*	28hpf: CTRMO vs. MCT8MO	ns	0.9177
		t=1.270, df=8 (Two-tailed)		30hpf: CTRMO vs. MCT8MO	ns	0.2397
		t=1.269, df=8 (Two-tailed)		32hpf: CTRMO vs. MCT8MO	ns	0.2400
		t=0.4882, df=8 (Two-tailed)		36hpf: CTRMO vs. MCT8MO	ns	0.6385
		t=4.299, df=8 (Two-tailed)		44hpf: CTRMO vs. MCT8MO	**	0.0026
		t=1.981, df=8 (Two-tailed)		48hpf: CTRMO vs. MCT8MO	ns	0.0830
		t=0.4744, df=8 (Two-tailed)		54hpf: CTRMO vs. MCT8MO	ns	0.6479
		t=0.5994, df=8 (Two-tailed)		72hpf: CTRMO vs. MCT8MO	ns	0.5655
**Supplementary Figure 2g**	Unpaired t-test	t=0.6199, df=8 (Two-tailed)	*robo4*	28hpf: CTRMO vs. MCT8MO	ns	0.5526
		t=0.6664, df=8 (Two-tailed)		30hpf: CTRMO vs. MCT8MO	ns	0.5239
		t=1.168, df=8 (Two-tailed)		32hpf: CTRMO vs. MCT8MO	ns	0.2764

		t=0.3422, df=8 (Two-tailed)		36hpf: CTRMO vs. MCT8MO	ns	0.7411
		t=0.2038, df=8 (Two-tailed)		44hpf: CTRMO vs. MCT8MO	ns	0.8436
		t=0.2180, df=8 (Two-tailed)		48hpf: CTRMO vs. MCT8MO	ns	0.8329
		t=1.130, df=8 (Two-tailed)		54hpf: CTRMO vs. MCT8MO	ns	0.2914
		t=0.1051, df=8 (Two-tailed)		72hpf: CTRMO vs. MCT8MO	ns	0.9189
**Supplementary Figure 2h**	Unpaired t-test	t=0.6232, df=8 (Two-tailed)	*slit2*	28hpf: CTRMO vs. MCT8MO	ns	0.5505
		t=0.4927, df=8 (Two-tailed)		30hpf: CTRMO vs. MCT8MO	ns	0.6355
		t=0.5680, df=8 (Two-tailed)		32hpf: CTRMO vs. MCT8MO	ns	0.5856
		t=0.8876, df=8 (Two-tailed)		36hpf: CTRMO vs. MCT8MO	ns	0.4007
		t=1.427, df=8 (Two-tailed)		44hpf: CTRMO vs. MCT8MO	ns	0.1914
		t=0.5495, df=8 (Two-tailed)		48hpf: CTRMO vs. MCT8MO	ns	0.5977
		t=1.399, df=8 (Two-tailed)		54hpf: CTRMO vs. MCT8MO	ns	0.1994
		t=1.363, df=8 (Two-tailed)		72hpf: CTRMO vs. MCT8MO	ns	0.2101
**Supplementary Figure 2i**	Unpaired t-test	t=0.2998, df=8 (Two-tailed)	*slit3*	28hpf: CTRMO vs. MCT8MO	ns	0.7720
		t=1.020, df=8 (Two-tailed)		30hpf: CTRMO vs. MCT8MO	ns	0.3375
		t=1.042, df=8 (Two-tailed)		32hpf: CTRMO vs. MCT8MO	ns	0.3278
		t=1.163, df=8 (Two-tailed)		36hpf: CTRMO vs. MCT8MO	ns	0.2784
		t=1.817, df=8 (Two-tailed)		44hpf: CTRMO vs. MCT8MO	ns	0.1068
		t=0.2483, df=8 (Two-tailed)		48hpf: CTRMO vs. MCT8MO	ns	0.8102
		t=1.528, df=8 (Two-tailed)		54hpf: CTRMO vs. MCT8MO	ns	0.1651
		t=2.255, df=8 (Two-tailed)		72hpf: CTRMO vs. MCT8MO	ns	0.0542
**Supplementary Figure 2j**	Unpaired t-test	t=0.2489, df=8 (Two-tailed)	*cxcl12b*	28hpf: CTRMO vs. MCT8MO	ns	0.8097
		t=1.283, df=8 (Two-tailed)		30hpf: CTRMO vs. MCT8MO	ns	0.2355
		t=0.6038, df=8 (Two-tailed)		32hpf: CTRMO vs. MCT8MO	ns	0.5627

		t=1.936, df=8 (Two-tailed)		36hpf: CTRMO vs. MCT8MO	ns	0.0889
		t=2.206, df=8 (Two-tailed)		44hpf: CTRMO vs. MCT8MO	ns	0.0584
		t=0.03133, df=8 (Two-tailed)		48hpf: CTRMO vs. MCT8MO	ns	0.9758
		t=0.3153, df=8 (Two-tailed)		54hpf: CTRMO vs. MCT8MO	ns	0.7606
		t=1.898, df=8 (Two-tailed)		72hpf: CTRMO vs. MCT8MO	ns	0.0942
**Supplementary Figure 2k**	Unpaired t-test	t=0.9008, df=8 (Two-tailed)	*cxcr4a*	28hpf: CTRMO vs. MCT8MO	ns	0.3940
		t=0.3869, df=8 (Two-tailed)		30hpf: CTRMO vs. MCT8MO	ns	0.7089
		t=0.8148, df=8 (Two-tailed)		32hpf: CTRMO vs. MCT8MO	ns	0.4388
		t=0.4766, df=8 (Two-tailed)		36hpf: CTRMO vs. MCT8MO	ns	0.6464
		t=1.906, df=8 (Two-tailed)		44hpf: CTRMO vs. MCT8MO	ns	0.0931
		t=0.05077, df=8 (Two-tailed)		48hpf: CTRMO vs. MCT8MO	ns	0.9608
		t=1.138, df=8 (Two-tailed)		54hpf: CTRMO vs. MCT8MO	ns	0.2879
		t=2.938, df=8 (Two-tailed)		72hpf: CTRMO vs. MCT8MO	*	0.0188
**Supplementary Figure 2l**	Unpaired t-test	t=0.9880, df=8 (Two-tailed)	*notch3*	28hpf: CTRMO vs. MCT8MO	ns	0.3521
		t=0.004462, df=8 (Two-tailed)		30hpf: CTRMO vs. MCT8MO	ns	0.9965
		t=1.660, df=8 (Two-tailed)		32hpf: CTRMO vs. MCT8MO	ns	0.1355
		t=0.4034, df=8 (Two-tailed)		36hpf: CTRMO vs. MCT8MO	ns	0.6972
		t=1.054, df=8 (Two-tailed)		44hpf: CTRMO vs. MCT8MO	ns	0.3225
		t=1.181, df=8 (Two-tailed)		48hpf: CTRMO vs. MCT8MO	ns	0.2716
		t=2.254, df=8 (Two-tailed)		54hpf: CTRMO vs. MCT8MO	ns	0.0542
		t=3.441, df=8 (Two-tailed)		72hpf: CTRMO vs. MCT8MO	**	0.0088
**Supplementary Figure 2m**	Unpaired t-test	t=1.503, df=8 (Two-tailed)	*s1pr1*	28hpf: CTRMO vs. MCT8MO	ns	0.1712
		t=0.3989, df=8 (Two-tailed)		30hpf: CTRMO vs. MCT8MO	ns	0.7004
		t=1.082, df=8 (Two-tailed)		32hpf: CTRMO vs. MCT8MO	ns	0.3109

		t=0.6042, df=8 (Two-tailed)		36hpf: CTRMO vs. MCT8MO	ns	0.5624
		t=0.8643, df=8 (Two-tailed)		44hpf: CTRMO vs. MCT8MO	ns	0.4126
		t=0.5995, df=8 (Two-tailed)		48hpf: CTRMO vs. MCT8MO	ns	0.5654
		t=1.120, df=8 (Two-tailed)		54hpf: CTRMO vs. MCT8MO	ns	0.2952
		t=0.9236, df=8 (Two-tailed)		72hpf: CTRMO vs. MCT8MO	ns	0.3827
**Supplementary Figure 4b**	CDA analysis	Mann-Whitney test (Two-tailed) U= 5	48hpf	*Tg(Pax8:dsRed)*+/- vs. *Tg(Pax8:dsRed)*-/-	****	<0.0001
